# Using micro-XRF to characterize chloride ingress through cold joints in 3D printed concrete

**DOI:** 10.1617/s11527-023-02132-w

**Published:** 2023-03-06

**Authors:** Paula Bran-Anleu, Timothy Wangler, Venkatesh N. Nerella, Viktor Mechtcherine, Pavel Trtik, Robert J. Flatt

**Affiliations:** 1 Nuclear Structures and Construction Group, Oak Ridge National Laboratory, Oak Ridge, TN USA; 2 Institut für Baustoffe (IfB), Physical Chemistry of Building Materials, ETH Zürich, Zurich, Switzerland; 3 TU Dresden, Institute of Construction Materials, Dresden, Germany; 4 Laboratory for Neutron Scattering and Imaging, Paul Scherrer Institut, Villigen, Switzerland; 5 Putzmeister Engineering GmbH, Max-Eyth-Str. 10, Alchtal, Germany

**Keywords:** 3D printing, Durability, Chloride, Transport, Neutron imaging, Cold joints

## Abstract

Digital fabrication methods with concrete have been rapidly developing, with many problems related to component production and material control being solved in recent years. These processes produce inherently layered cementitious components that are anisotropic, and in many cases, produces a weak interface between layers, which are generally referred to as cold joints. While material strength at these interfaces has been well studied in recent years, durability has received less attention, even though cold joints can function as channels for aggressive agents, such as chlorides. This work presents a method using micro-X-ray fluorescence (*μ*XRF) to image chloride ingress into layer interfaces of 3D printed fine-grained concrete specimens produced with varying layer deposition time intervals, and also compares it to neutron imaging of moisture uptake. The results show that cold joints formed after a 1 day time interval are highly susceptible to chloride ingress, and that curing conditions play a major role in how quickly interfacial transport can take place. The *μ*XRF method is also shown to be useful for study of transport of chlorides in cold joints, due to its spatial resolution and direct analysis of an aggressive species of interest.

## Introduction

Digital fabrication signifies a revolutionary step in manufacturing [1]. In recent years, it has been gaining more and more popularity in the manufacturing field, as it provides a relatively convenient technique to apply digital modeling and technologies to the production of custom objects on demand [2–5]. One of the newer and fast growing applications of digital fabrication in the recent years has been in the field of architecture and construction; since in addition to lower costs for customization, it also promises faster construction, lower costs in terms of labor and formwork, while improving worker safety [6–11]. This is because the technique allows for a more precise material placement and more efficient construction practices. Moreover, absence/minimization of formwork contributes to a reduction in waste generation in the construction field. This is of particular interest in the adoption of more material efficient structural shapes, which until now has been inhibited from more widespread adoption by the use of costly customized formwork systems.

The above described technique has also been called digital construction [12]. By far, the most popular technique in research and industry has been extrusion-based 3D printing, also called 3D concrete printing (3DCP). It is a scaled up version of fused deposition modeling (FDM), which is the deposition of a filament of extruded material from a digitally controlled printhead, layer-upon-layer. This technique has been reviewed in a number of recent publications [12–15]. This layered process (from here referred to simply as 3D printing) is well-established in fields such as aerospace and medicine [2], that works well for polymeric materials in which material is extruded in the liquid or plastic state that later hardens, analogous to concrete. Although 3D printing of polymers and polymer composites has undergone significant developments in recent years, several limitations still prevail [2]. First of all, material properties and their evolution over time play a significant role in the ahility to successfully 3D print an object. Secondly, the technique also introduces anisotropic mechanical properties due to voids that can be present at the layer-to-layer interfaces of 3D printed components. Third, the manufacturing of the appropriate printing machines poses another limiting factor. These limitations and challenges are of course inherited by 3D printed concrete, and as a consequence they are being widely investigated along with 3DCP own limitations and challenges specific to cementitious materials [2, 6, 7, 12, 13, 16–20].

Although it is true that digital fabrication in the construction field has great potential, some questions are still open and are yet to be thoroughly explored. One aspect that, while somewhat ignored at first, is the effect of the quality of the layered concrete interfaces, although in the past half decade it has received much more attention. Le et al. [7], Nerella et al. [21], Panda et al. [22], and Wolfs et al*.* [23] are some of the first researchers to investigate the formation of weak interfaces (also referred to as cold joints) between printed layers and their effects on the mechanical performance of 3D printed cementitious materials, a topic now more recently investigated and reviewed by others [20, 24–26]. These studies confirmed that material composition and time intervals between placements of subsequent layers showed an obvious influence on the bond strengths of the interfaces, which in turn influenced the mechanical properties of the material [21]. Furthermore, Zareiyan et al. [27] and Panda et al. [28] studied the influence of aggregate size and process parameters on interlayer adhesion, respectively. The moisture content of the layer-to-layer interface region, and in particular the moisture on the substrate layer, has been identified as one of the “major factors affecting the inter-layer strength” [23–25]. Specimens which are exposed to drying at room temperatures lead to weaker interface bonds in comparison to those which are protected from drying [23, 24]. It must be here noted that it is not always possible to protect 3D printed concrete structures from drying, especially in large scale and/or on-site 3D concrete printing. A final source of poor interlayer quality is the potential to entrap voids during printing, which has also been noted [29].

The time intervals between layers are finite and variable depending on printing speed and contour length [30, 31]. This, together with the material structuration rate and/or the loss of moisture, potentially has a clear effect on the interface between layers [6]. This was proven in the above cited literature, in terms of the mechanical properties of 3D printed concrete. However, the importance of such phenomenon and its impact on durability remains an open question. Chloride permeation leading to chloride-induced corrosion is one of the primary concerns in concrete durability, and interfaces are known to change behavior from the bulk properties of concrete, based on concrete literature examining mechanical properties across interfaces [32, 33]. Explicitly for 3D printing concrete, Van der Putten et al. have examined interfacial microstructure, and have also shown that chlorides can penetrate through material interfaces at higher speeds than in the bulk depending on interlayer time gap, using a silver nitrate and image analysis method [26, 34, 35]. The same authors also performed a separate neutron imaging study in continuously printed interfaces (short interval times) and found no preferential moisture ingress [36]. As previously mentioned, the material composition is known to have a strong influence on concrete’s durability [37–42]. In the case of digital fabrication, the sensitivity to the material is probably exacerbated and is coupled to factors such as time interval and moisture content. Thus, on top of concrete’s identifiable issues in terms of durability, the additional effect of formation of cold joints needs to be addressed and investigated for digital fabrication.

In this study, the issue of layer interfacial effects on moisture and chloride ingress is studied in a combined study of neutron imaging and micro X-ray fluorescence. Neutron imaging is a useful and well known technique to study moisture transport in concrete [43], and micro X-ray fluorescence is an elemental analysis technique often applied in cultural heritage studies [44] and gaining more use in concrete material science [45, 46]. For this study, the ingress of chlorides is qualitatively compared to moisture ingress in samples 3D printed with variable interlayer times, and the advantages and disadvantages of both methods at determining the existence of a cold joint are discussed. This study highlights the usefulness of a method with good spatial resolution [46] to investigate cold joint formation in a relevant case. Chloride ingress is observed in 3D printed fine-grained concrete specimens produced at the Institute of Construction Materials, TU Dresden. A parallel study of moisture ingress was conducted as well on larger scale samples and is reported in reference [47], however the work in this study is aimed at analyzing and comparing chloride ingress to the neutron imaging method.

## Materials and methods

### Materials for the 3D printed samples

Portland cement (CEM I 52.5 R according to EN 197-1) was provided by OPTERRA Zement GmbH, Karsdorf, Germany. Very fine quartz sand (BCS 413, 0.06–0.2 mm) provided by Strobel Quarzsand GmbH, Germany and two fine natural river sands (0–1 and 0–2 mm) from Kieswerk Ottendorf-Okrilla GmbH & Co. KG, Laußnitz, Germany complying with EN 12620/13139 [25] were used as aggregates. Micro silica suspension (50 wt% aqu. Suspension, EM-SAC 500 SE, Elkem), fly ash (class F, Steament H-4, STEAG), and a high range water reducing admixture (Gelnium Master SKY 593, BASF) were also used. The mix proportions are presented in Table 1. Particle size distributions of the aggregates can be referred to in [23].

**Table 1:** Mix proportion for all 3D printed samples (kg/m^3^)

Material	Content
Portland cement	378.4
Micro silica suspension	206.4
Fly ash	206.4
Fine sand (0.06–0.2 mm)	316.3
Sand (0–1 mm)	278.0
Sand (0–2 mm)	717.3
Water (*w/c* = 0.42)	133.7
Water reducing admixture	10.32

### Materials for chloride exposure

Sodium chloride (NaCl for analysis EMSURE ®, ≥ 99.5%, ACS ISO, reag. Ph. Eur., Merck KGaA, Darmstadt, Germany), was used as chloride source. Sodium hydroxide (Sodium hydroxide pellets, analytical reagent grade, conforming to BP, EP; ≥ 98.3%; Fisher Chemicals, New Hampshire, USA), and calcium hydroxide (calcium hydroxide for analysis, puriss. p.a., Reag. Ph. Eur., 96%, Sigma-Aldrich, Missouri, USA) were used for the chloride solution to which the samples were exposed.

### Preparation of 3D printed samples

**Fig. 1:**
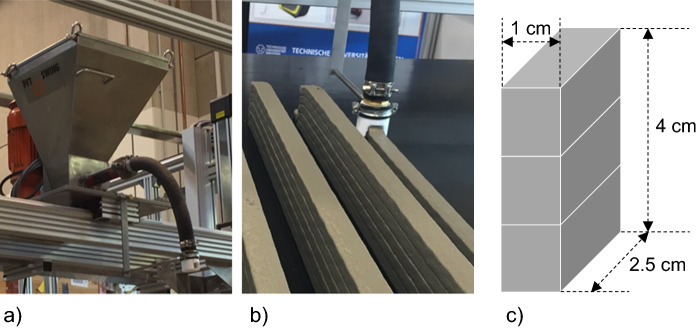
**a** Printhead of the 3D-printing test device **b** sample wall specimens and **c** schematic of extracted specimens for the chloride ingress test; the long face (4 by 1 cm^2^) is exposed to chlorides and moisture ingress

3D printing of specimens was carried out at the Institute of Construction Materials, Technische Universität Dresden, using the 3D printing test device (3DPTD), prototype 1. The 3DPTD, is a multi-axis portal system with stepper-motor driven printhead (see Fig. 1a) that can be moved within a working area of approximately 3 × 1.5 × 1.5 m^3^. The nozzle (see Fig. 1b) used is made of polyoxymethylene and has dimensions of 3 cm by 2 cm. Additional details for the 3DPTD can be referred to in [21].

**Fig. 2:**
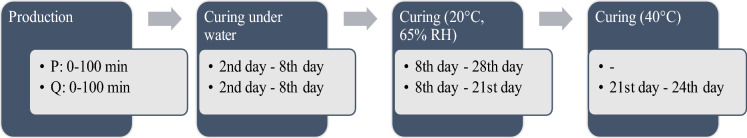
Graphical representation of the curing process for the printed specimens. Process P has no additional drying conditions during the last week, whereas process Q includes a 3-day drying condition at 40 °C

Printable fine-grained mortar (from now on refered to as concrete) of volume 35 l was produced with a single-shaft pan mixer; see [21] for the mixing regime. The water-to-cement ratio for all specimens was 0.42. Beginning at the concrete age of 20 min, three layered walls were printed with total wall dimensions of 120 × 3 × 6 cm^3^. These walls were cured under water from 24 h to 7 days. After which, they were stored in a climatic chamber (20 °C, 65% RH) until the age of 20 days. At the age of 20 days, specimens of dimensions 4 × 1 × 2.5 cm^3^ were obtained from the large walls by wet cutting. Each cut specimen contains 2 interfaces (3 layers) along the 4-cm edge (see Fig. 1c). From day 21 until day 24, some of the specimens were cured in an oven at 40 °C (group Q) whereas the other specimens were stored in a climatic chamber (20 °C, 65% RH); see Fig. 2.

Three groups of samples were prepared, where the printing interval was varied to study its effect. The first and second groups have 2- and 13-min interval between layers. The third group has a 2-min interval for the first interface (1st layer–2nd layer) and a 24-h interval for the second interface (2nd–3rd layer). A summary of the time intervals for each sample and sample notations are presented in Table 2.

**Table 2:** Summary of sample classification

Sample	Interface time interval (min)	Curing conditions
A29	13	P
A30	13	P
A35	13	Q
A36	13	Q
A40*	2	P
A44	2	P
A45	2	P
A48*	2	Q
A50	2	Q
A51	2	Q
A55*	1440	P
A59	1440	P
A60	1440	P
A62*	1440	Q
A65	1440	Q
A66	1440	Q

For each sample the time interval between interfaces and the curing conditions are given. Condition P has no extra drying conditions during the last week, whereas condition Q includes a 3-day drying condition at 40 °C at the end. Samples with * were used in neutron imaging studies, and all other samples tested for chloride ingress. Sample number is only a label

It is important to underline that the third stage of curing at 20 °C and 65% RH also should be considered as a drying stage leading to a loss of capillary water. This is important for what follows as the samples are not further dried before capillary rise experiments for chloride ingress or water mapping by neutrons.

### Chloride exposure and imaging by μ-XRF

#### Exposure experiments

**Fig. 3:**
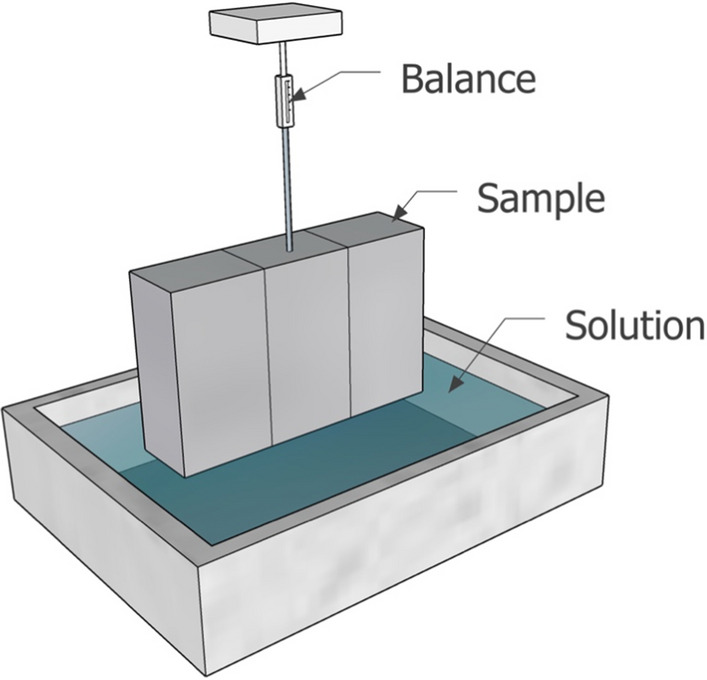
Schematic representation of the surface exposed to chlorides, and a similar orientation was used in neutron radiography experiments. For chloride exposure, the samples were suspended from a balance that recorded the mass increase of the specimen resulting from absorption of the solution. No preconditioning was performed on the specimens prior to chloride exposure. For neutron radiography, there was no suspension from a balance, and the sample was placed on two small wires, with water filled just touching the bottom of the sample, and the detector just behind the back face of the sample

The samples were exposed to chlorides by means of capillary rise. They were not further dried than as received, since the curing steps described include such a stage. The experimental setup is basically the same as for a sorptivity (capillary moisture uptake) experiment. The samples were suspended from a balance, and one of the faces with the two interfaces was in contact with a solution containing the chlorides schematically shown in Fig. 3. The exposure solution consisted of 1 M NaCl + 0.1 M NaOH + sat. Ca(OH)_2_. The experiment was run for 24 h, after which the samples were dry cut perpendicularly to the exposed surface. Stopping the experiment and cutting the sample was done the morning right before starting the micro XRF mapping.

#### Micro X-ray fluorescence

Micro X-ray fluorescence (µXRF) is an elemental analysis technique which allows for the examination of relatively small sample areas. Unlike conventional XRF, which has a typical spatial resolution ranging from several hundred micrometers up to several millimeters, µXRF uses polycapillary optics to generate small focal spots with high X-ray flux on the sample surface with a spatial resolution on the micrometer scale [48–50].

In the last three decades, *μ*-XRF analysis has become more easily accessible for routine analysis. The selection of the incident beam energy is in the range of 15–40 kV, which provides the excitation of at least one measurable characteristic X-ray peak for all elements of the periodic table with an atomic number greater than 11 (sodium) in various media. The said spectra can be collected either in low vacuum or at atmospheric conditions. The method allows a high flux of photons, adjustable wavelengths and advanced X-ray focusing techniques, which are needed for high spatial resolution chemical imaging. The X-ray beam is excited using a rhodium (Rh) target, and this allows the detection of major and minor constituents of complex materials (e.g. cement paste) [49, 50].

In this study, the instrument that was used for the *μ*XRF analysis is an EDAX (Mahwah, NJ, USA) ORBIS *μ*XRF spectrometer. The system uses a Silicon Drift Detector (SDD) and focuses the X-rays from a rhodium target anode with a polycapillary focusing optic, which allows for a beam diameter of roughly 30 μm (FWHM at molybdenum Kα line, 17.5 keV). The SDD has an active area of 30 mm^2^ and an 8 μm beryllium window with an energy resolution of less than 165 eV at manganese Kα line (5.9 keV). The acquisition system is the ORBIS Vision Software by EDAX.

The measurement protocol was the following: The relatively freshly cut surfaces of the samples were mapped with a 45 μm resolution. The applied acceleration potential and current were 35 kV and 950 μA, respectively. At each point, a spectrum was acquired for 150 ms. Measurements were carried out in air with a built-in 25-μm thick aluminum filter to eliminate the rhodium Lα radiation and, therefore, preventing it from reaching the sample. This is necessary because this radiation overlaps with the chlorine Kα X-ray line and increases its limit of detection.

### Moisture ingress by neutron imaging

Similarly to the chloride ingress, samples were not further dried than as received, since the curing steps described include such a stage. Samples from the same sample group were investigated at the NEUTRA thermal neutron imaging beamline, at the SINQ spallation neutron source in the Paul Scherrer Institute, Villigen, Switzerland [51]. The high neutron cross section of hydrogen allows for visualization of the distribution of the hydrogenous materials in cementitious materials (here H_2_O). A protocol similar to that presented in [52] was used, except measuring position 2 was used, with a field of view of 15 × 15 cm^2^, for higher resolution of smaller samples [53]. Samples were oriented similar to Fig. 3, with moisture ingress occurring parallel to the layer interfaces, and the neutron beam passing through front face of the sample and striking the detector behind. the investigated for 3–8 h for each individual sample, determine primarily by beam stability. In any case, these durations are generally adequate to see large differences in capillary sorption of moisture along these layer interfaces. Four samples were imaged during the available beamtime due to the inconsistency of the neutron beam: those corresponding to the 2-min layer interval and the 24-h layer interval, with both curing conditions, as referenced in Table 2. Only these samples were imaged due to beamline availability issues limiting sample choice to just extreme cases. Details of the measurement setup and post-processing are provided in the following section.

#### Neutron imaging and analysis

The samples were held within an aluminum receptacle (as aluminum is comparatively transparent to neutrons), laid on two small wires. The sample and frame were placed in measurement position 2 at the NEUTRA beamline [53]. After traversing the sample the neutron beam was detected by scintillator-camera type detector. The 20 µm thick gadolinium oxysulfide scintillator screen (RC Tritec) together with sCMOS (Andor Neo) camera were used in this investigation. The pixel size of the acquired images was equal to 15.7 µm. The spatial resolution of this imaging system as assessed by the gadolinium Siemens star in contact with the detector test object was equal to approximately 40 µm. The samples were first imaged with no moisture intake for a reference; approximately 10 images were taken. Then an automatic titration system deposited water in the aluminum receptacle until the water reached the bottom of the sample, and the wetting experiment started. During these experiments, images were taken every 12 s.

Images were analyzed in the open source image analysis program ImageJ [54]. All samples had 5–10 dark current images (shutter closed) and 50 open beam images (shutter open, without sample), and an average image for these two conditions was taken. Before any analysis took place, all raw, uncropped images were treated to reduce noise by first removing bright outliers, then using the despeckle feature in ImageJ to smooth out any other noise as much as possible. The first and last 10 images of the sample were then averaged to form dry and wet reference images, respectively. Using the Image Referencing plugin in ImageJ [55], the dry and wet reference images were converted into transmission images through normalization by the open beam image, after subtraction of the dark current image. The difference between the wet and dry transmission images were then taken. Within this time frame, it was adequate to qualitatively determine if there was preferential uptake along the layer interfaces.

## Results

### Chloride maps

**Fig. 4:**
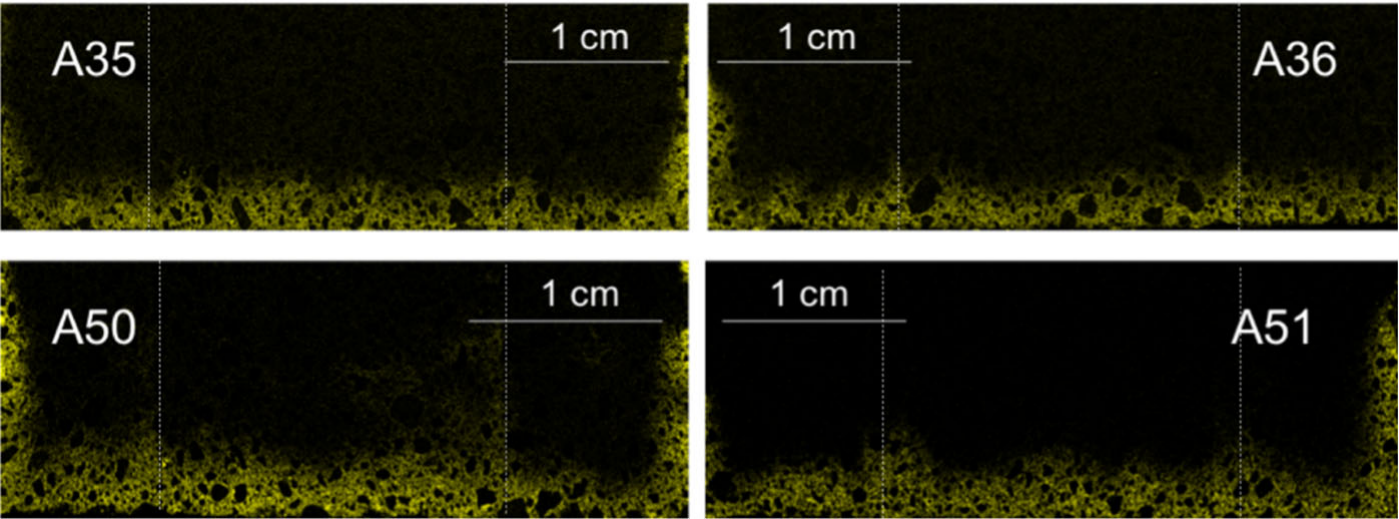
Micro XRF chlorine maps for samples A35, A36, A50 and A51 (curing condition Q). The bottom of each image represents the direction from which the chloride solution penetrated. Each map has a slightly different size, thus each of them has its own scale. The dotted lines represent where the interface between layers is

**Fig. 5:**
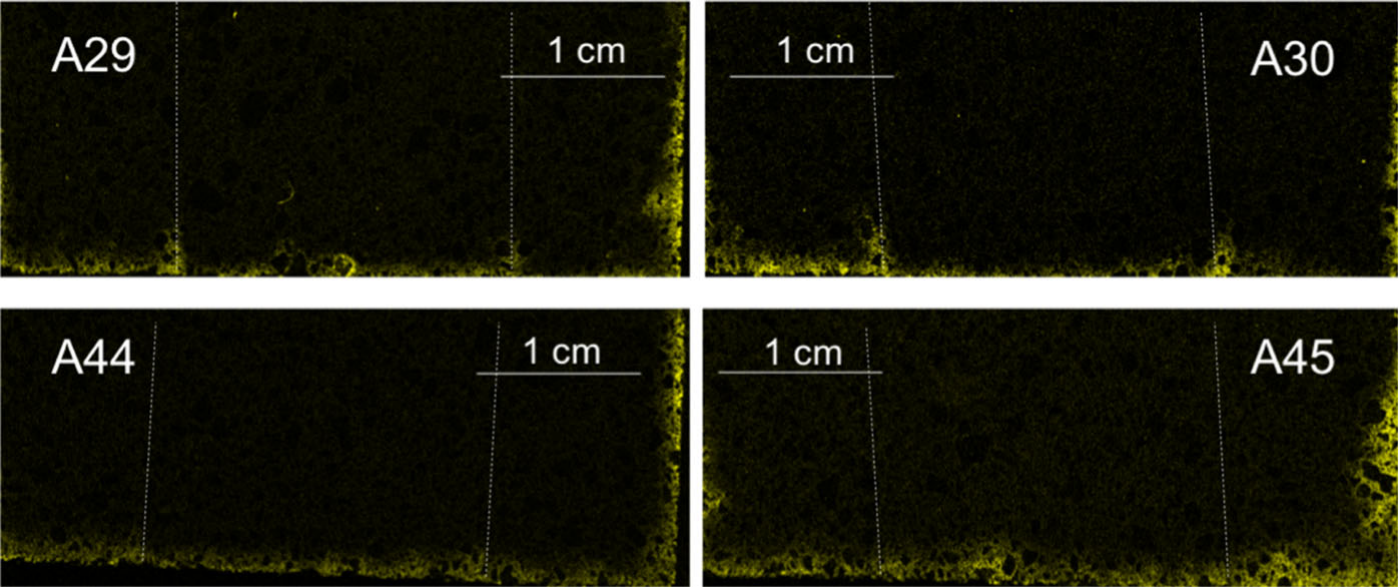
Micro XRF chlorine maps for samples A29, A30, A44 and A45 (curing condition P). The bottom of each image represents the direction from which the chloride solution penetrated. Each map has a slightly different size, thus each of them has its own scale

Chlorides maps were collected for all samples, however due to time constraints, not all maps are of the same size. In order to keep the same resolution for all maps, some maps needed to be smaller than others. Still, if it deemed necessary, the area of the map was chosen to include all regions of interest. Figures 4 and 5 show the chloride maps for samples A29 through A51, corresponding to curing conditions Q and P, respectively (also refer to Table 2 and Fig. 2).

As seen in Fig. 4, no clear cold joint is visible even though the layer interfaces are somewhat recognizable in some of the samples. Another visual evidence is the difference in chloride penetration depth (Figs. 4 and 5). In Fig. 5, the penetration depths are visibly smaller than for the samples in Fig. 4.

**Fig. 6:**
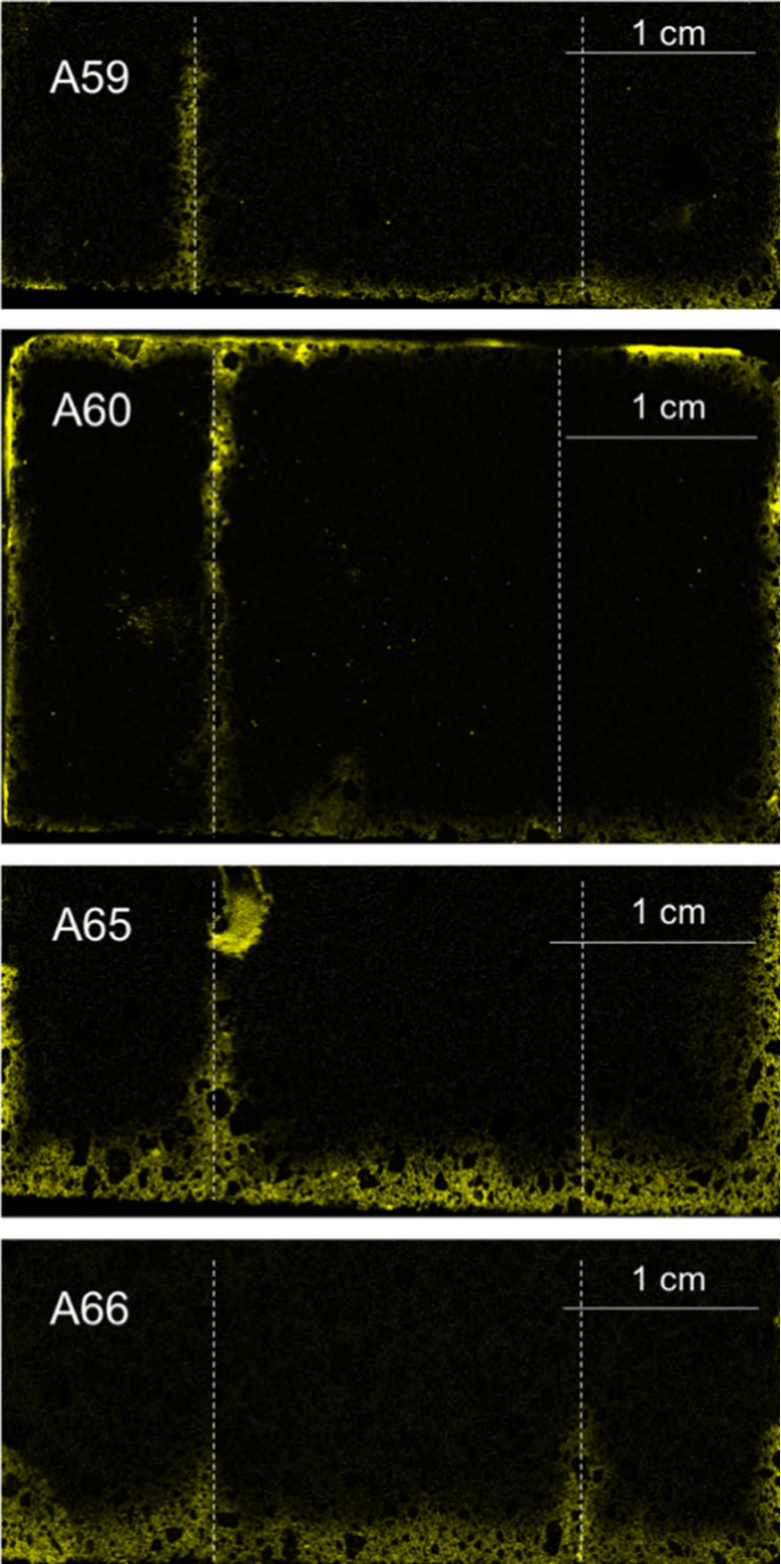
Micro XRF chlorine maps for samples A59 through A66. The bottom of each image represents the direction from which the chloride solution penetrated. Each map has a slightly different size, thus each of them has its own scale. Interlayers indicated by dashed lines

**Fig. 7:**
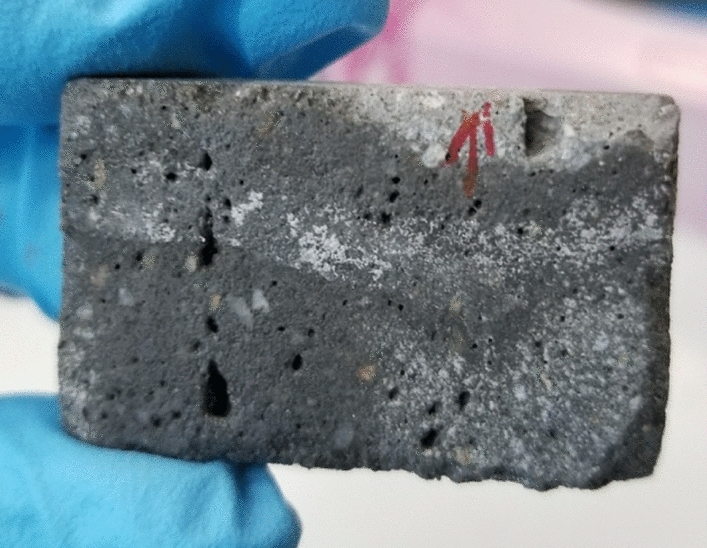
Efflorescence (white stains) in sample A60, as it was after the sorptivity experiment was stopped. The red arrow indicates the top of the sample. After visual inspection, it was decided to make a chemical map of the entire sample

Figure 6 shows the chlorine maps for the rest of the samples. These samples represent the four cases in which one of the layered-interface had a time interval of 24 h. These samples show the worst conditions in terms of penetration depth of chlorides. Also, apart from sample A66, they all exhibit a clear cold joint. Sample A65 even contained a large cavity in the interface to the left, which was not visible until the sample was cut, and sample A60 shows by far the worst conditions. The chloride solution went all the way through the cold joint, and it showed some efflorescence after the experiment was stopped (see Fig. 7).

### Neutron imaging of moisture

**Fig. 8:**
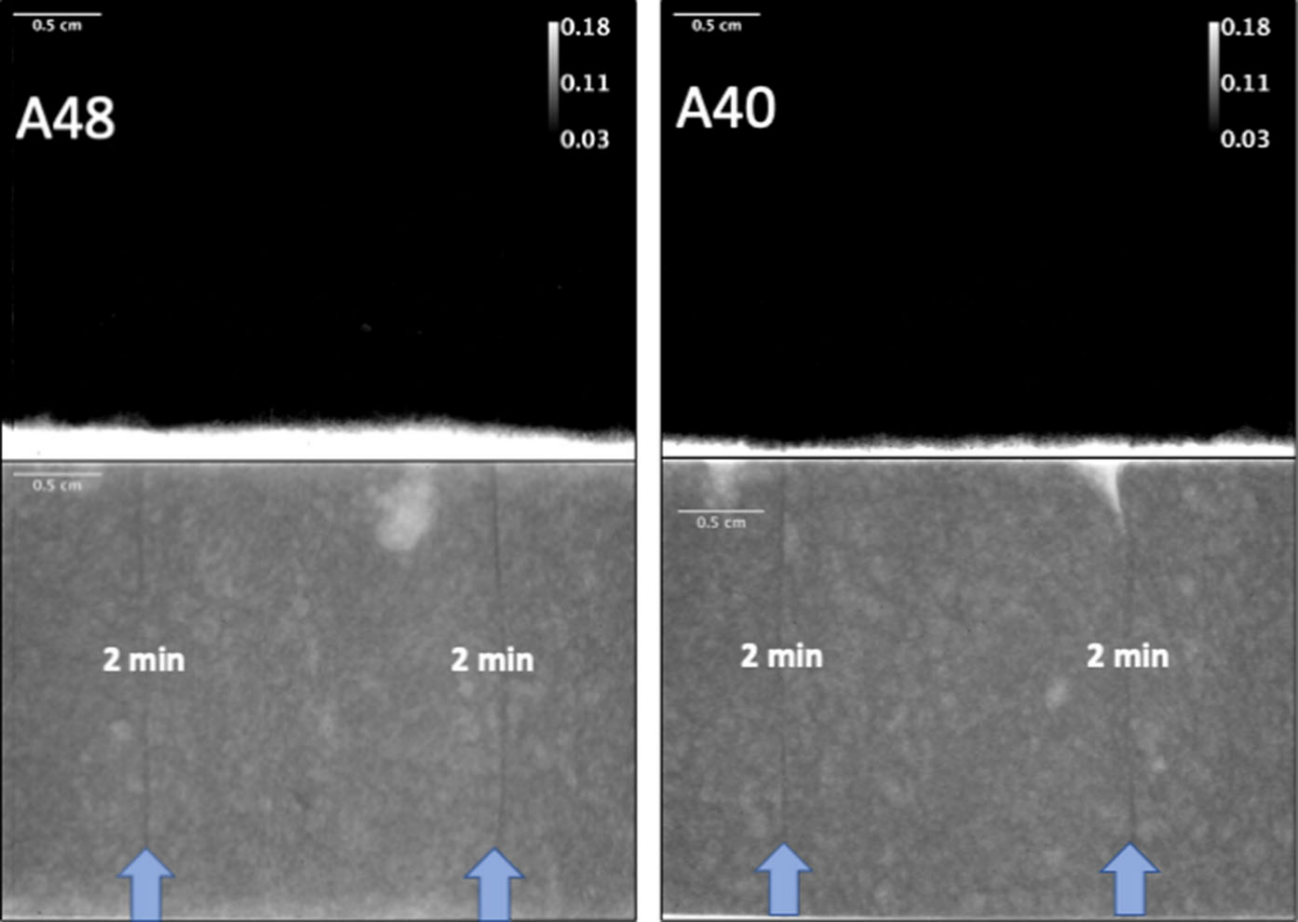
Transmission image difference profiles (top) and dry reference images (bottom) for samples A48 (left, wet curing condition “P”) and A40 (right, dry curing condition “Q”). White areas in transmission difference profiles correspond to areas of high moisture ingress, with gray value scale bar added for information. Blue arrows on dry reference images indicate location of layer interfaces, and time interval between layers is indicated on image. Scale bars are equal to 0.5 cm on all images

**Fig. 9:**
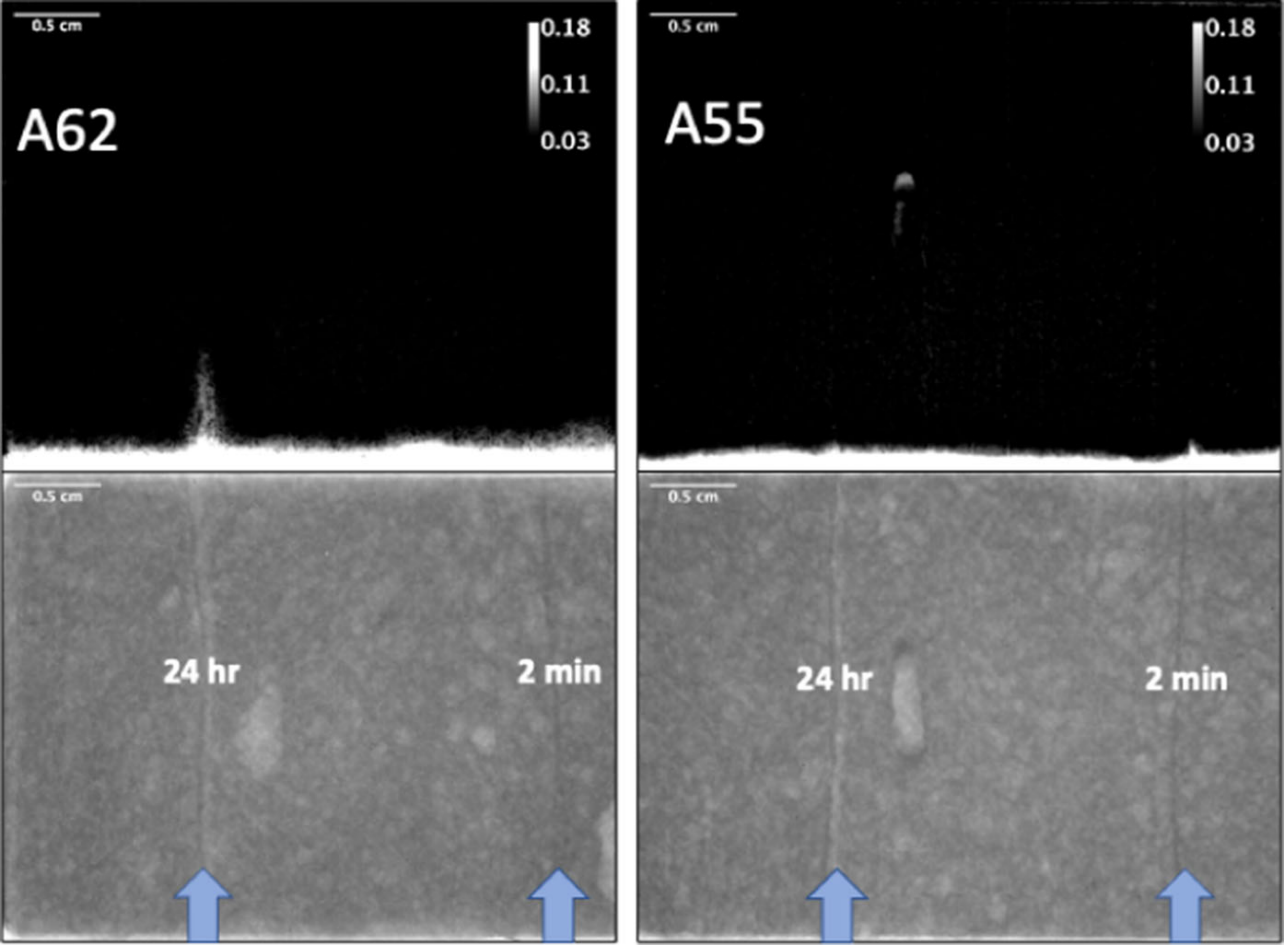
Transmission image difference profiles (top) and dry reference images (bottom) for samples A62 (left, wet curing condition “P”) and A55 (right, dry curing condition “Q”). White areas in transmission difference profiles correspond to areas of high moisture ingress, with grey value scale bar added for information. Blue arrows on dry reference images indicate location of layer interfaces, and time interval between layers is indicated on image. Scale bars are equal to 0.5 cm on all images. Sample A55 image difference has an image artifact in the center where moisture condensed in a large void (visible in dry reference image) during the measurement

Figures 8 and 9 give qualitative results of the moisture ingress. Each set of images shows the dry reference image for a particular sample on the bottom, and the projection difference image on the top. Figure 8 corresponds to both curing conditions for samples with only a 2-min delay, and Fig. 9 corresponds to both curing conditions for the samples with the 24-h delay (on one interface, the other interface had 2 min delay).

One can observe from these images that only the 24-h delayed sample that was cured in an environment where it could dry out showed significant moisture ingress along the layer interface. This is in correspondence with [52]. It is also worth noting that one can observe in all the dry reference images that layer interfaces are easily seen as dark lines, indicating a higher level of water. This is most likely due to their paste rich nature, where the lubrication layer of the extruded filament has higher paste content, thus higher amount of hydrates. In the samples with a 24 h layer interval time (Fig. 9) one can also observe at the 24 h layer interface a lighter region next to the darker layer interface. This lighter region corresponds to the top of the printed interface that was exposed to drying conditions for 24 h, and is thus lower in moisture content compared to the bulk.

## Discussion

An earlier study [21] clearly indicated that interfaces are present between layers of 3D printed concrete or mortar specimens. It was more evident in some cases than in others, but generally depended on the time intervals between the placement of subsequent layers. Hence, the time interval has a significant influence on the quality of the interfaces or the bonds between layers, with a general relationship showing a reduced quality at longer time intervals [7, 20, 21, 23, 25, 26, 34, 56]. This reduced quality is usually shown in lower strengths and/or increased permeability. Some microstructural explanations have been put forth, such as higher porosity in the region of the interlayers, although the microstructural explanations are beyond the scope of this work.

In this particular study, the significance of the influence of the time interval between layers on the penetration depth was not as strongly evident as the influence of the curing conditions. The additional 3-day drying period at 40 °C in curing condition Q, made a difference in the depth of chloride and moisture ingress. This is of course not a surprise, as the drying condition would remove more water from the pores than the 65% curing to which the other samples were exposed, thus explaining the increase in the capillary rise of the Q-cured samples.

The results of the neutron imaging experiments partially corroborate the results of the chloride maps, but seemingly with less resolution. Only the 24-h interface with an accelerated drying curing condition showed any significant differential moisture penetration, whereas the chloride ingress experiments were able to image chlorides entering in some interfaces with shorter time intervals. The fact that the chloride experiments lasted for 24 h, versus 3–8 h for the moisture transport, could have been a factor. This is also combined with the fact that chlorides have two driving forces for transport into the sample: initially, convective transport driven by capillary absorption followed by diffusive transport once the sample is saturated. For the neutron imaging experiments, only capillary absorption is the driving force. In any case, the chloride mapping with *μ*XRF method seems more sensitive to detect transport in interfaces, probably due to the fact that the neutron imaging method requires a certain amount of differential moisture content across a particular cross section to ensure that an appropriate signal can be seen, and this could be smaller than that required for the chloride mapping method. Also, the chloride mapping method is a bit simpler to perform, and with less expensive equipment, all the while imaging a species that is of chief concern in concrete durability. However, it should be noted that the chloride *μ*XRF method is a destructive method, and loses the advantage of time resolved measurements that neutron imaging has, and thus the ability to observe processes in situ.

**Fig. 10:**
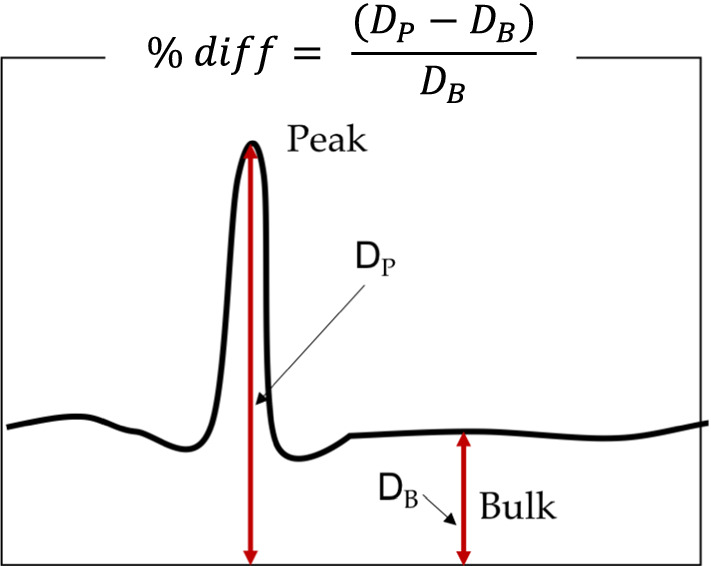
Sketch of a typical chloride ingress pattern where the ingress at joints are visually different than the bulk ingress

**Fig. 11:**
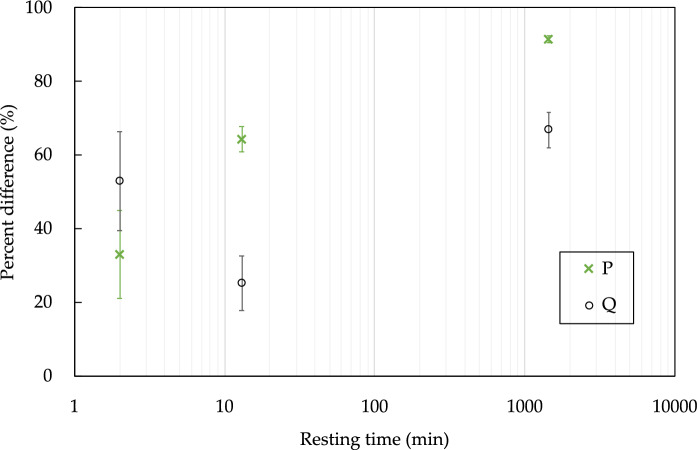
The percent difference between interface penetration depth and the bulk penetration versus the time interval between layers. The error bars represent the standard deviation. The ‘x’ symbols represent the samples with curing condition P (curing/drying at 65% RH and 20 °C), and the hollow circles are the samples with curing conditions Q (extra 3 day drying period)

There was however a somewhat contradictory result in terms of the effect of the time interval on the chloride ingress. If the samples with curing condition Q in Fig. 4 (A35, A36 and A50, A51) are examined, even though the samples show a considerable depth of penetration, the interfaces are less visible for samples with a longer interlayer time interval (A35, A36). If on the other hand, the samples with curing condition P, (A29, A30 and A44, A45 in Fig. 5) are examined, the opposite can be observed. The penetration depth is small in both cases, but the samples with a longer time interval (A29, A30) show a more pronounced evidence of the interfaces between layers. This occurrence could perhaps be explained by the discoveries of Sanjayan et al. [25], where they reported that, in contrast to [7], interlayer strength does not necessarily decrease with increasing time interval, but it depends more on the amount of moisture on the surface of each extruded layer. They reported that (for their particular mix) the moisture level on the surface first decreased, and then increased with increasing interval times. This was also observed for the interlayer bond strength. It is of course also possible that printing and material parameters can cause higher porosities and air void inclusion at the interface as well, which certainly can also be affected by the curing conditions. In any case, though more analyses are required to corroborate this principle for the particular mix under investigation, as a first approximation the difference of the penetration depths was estimated using the chloride maps. The percent difference, or penetration enhancement, was calculated as seen in Fig. 10. The calculation was done for each peak. Ten measurements were made for each depth in order to obtain an average value for *D*_P_ and *D*_B_. The results of this calculation are presented in Fig. 11.

**Fig. 12:**
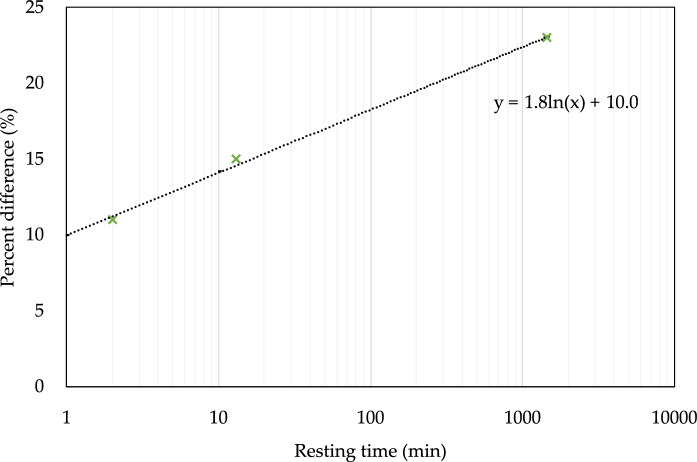
Flexural strength loss due to layered manufacturing given as the ratio of the difference in flexural strength normal and parallel (to interface) to the flexural strength in the normal direction, as a function of time gaps (or time intervals). Samples were tested at age of 28 day. Data from [21]

As can be seen from Fig. 11, the percent differences are more scattered for the samples with a 2-min time interval than for the 13-min interval. This suggests that there is something more than just the time interval that affects the difference in the penetration depths. Additionally, the curing conditions have a significant effect for samples with a 13-min interval. While the drying condition at the end of curing leads to larger penetrations depths, the difference between the bulk and the layered interfaces is not so pronounced. One expects permeability and strength to be inversely related, and Nerella et al. [21] performed strength tests on similar samples to the ones being investigated here (with a curing regime similar to “P”), and they found that the flexural strength difference (calculated as the difference between the flexural strength loading normal to the interfaces and the flexural strength loading parallel to the interfaces, normalized by the flexural strength loading normal to the interfaces) does not linearly increase with increasing time interval, but it is rather a logarithmic relationship (see Fig. 12). The time intervals are too far apart for us to directly compare to the results of [25], however the non-monotonic behavior does suggest that there is something other than simply the time interval at play.

Another interesting finding is the effect of a cold joint on the resistance of concrete to chloride penetration. As it can be clearly seen from Figs. 6 and 7, sample A60 was the one with the worst performance in terms of chloride and moisture ingress. This shows that the time interval between layers is finite and needs to be controlled as was discussed in [6]. Likewise, it can be seen in Fig. 11, where the 24-h time interval leads to almost 100% difference in the penetration depths. Another important point to highlight is how the percent differences in Figs. 11 and 12 compare. Looking at the strength loss values, the extent of reduction in flexural strength is much lower than the chloride penetration enhancement, where for a resting time of 24 h it is only about 23% compared to 90% enhancement for the corresponding samples. This confirms that the large time interval between layers could be detrimental for a concrete object or structure in terms of its durability. This is important, not only for chloride ingress, but also for other substances that can damage the concrete or reinforcement, such as sulfates and CO_2_.

Furthermore, since the possible formation of cold joints in 3D printed structures creates an imminent threat on the structure’s durability, it is even more important to have access to a method that offers a spatial distribution of the aggressive agent, chlorides for example. For one thing, if the samples in Fig. 6 were being analyzed for chloride content at a certain depth, the results with the conventional methods would certainly be misrepresentative. Like it can be seen in Fig. 6, the penetration depth at the cold joint is much larger than in the rest of the sample. Henceforth, conventional methods used in practice to characterize chloride profiles do not work in this case. An average value over cross-sections is extremely misleading for the case of cold joints in 3D printed concrete.

It has been shown that chloride induced pitting corrosion in reinforced concrete has a stochastic nature, and that already in regular concrete it poses a misleading notion in terms of the critical chloride content at the concrete-steel interface for corrosion initiation [57–59]. Angst et al. [57] stated that local chloride concentration as well as interfacial or defects on the surface of the reinforcement, all exhibit spatial variability, and that depassivation of the reinforcement only occurs by coincidence of the latter with a sufficiently high local chloride concentration. On top of this, the front of the chloride ingress in a concrete sample is not uniform, even if the parameters of exposure are controlled in a laboratory [58]. With all this being true for regular mold-cast concrete, the need for a spatial resolution of chloride ingress is only heightened in the case of 3D printed concrete. Without chloride mapping in 3D printed structures, one would all too often conclude that chloride concentrations are not critical, while the contrary may be true.

Finally, it is also important to note that all samples examined in this study had a 7-day wet curing after production. This represents a quite advantageous situation with respect to what can be expected for structures produced by 3D extrusion printing that for the most part is directly exposed to drying at atmospheric conditions. Thus, the fact that these samples mark the issue of cold joints implies that this issue is probably even greater for normal extrusion printing which would normally not benefit from a 7-day wet curing.

## Conclusions and outlook

Although there is great potential for 3D printing of concrete to provide significant contributions to the construction industry, there are still several challenges that need to be addressed. As it was reported in other studies [7, 21, 25, 52], and supported by the work done for this study, an important challenge is the bond quality of the interfaces between layers of a printed concrete or mortar, as it can have a direct impact on the strength. It is important to understand this, not only to improve mechanical properties of the printed objects, but ultimately for durability issues as well.

Overall, it was confirmed that the curing conditions play a very important role in the quality of the interfacial bond between layers, especially in terms of moisture and chloride ingress into the material. The role of the time interval between layers is slightly less straightforward. Although it is evident that 24 h delay time is not at all ideal, the difference between 2 and 13 min for the studied mix is not so obvious. Sanjayan et al*.* [25] indicated a dependence on surface moisture on the previously extruded layer. Clearly, more detailed analyses are needed to clarify this phenomenon, and in particular a close examination of time intervals that could reflect real processing conditions and delays, somewhere between the 13 min and 24 h of this particular study.

On the whole, the presence of cold joints proved to be a detrimental aspect for a 3D printed concrete or mortar object in terms of its durability. Whether it is chloride induced corrosion (in case there is steel reinforcement present) or other substance attack (e.g. sulfate attack), a cold joint has little to no resistance to moisture penetration compared to the bulk. Furthermore, using conventional methods to characterize the chloride profile over a cross-section, for example, is extremely misleading and so a method that offers a spatial resolution, such as that presented in this article, is highly needed.

Implications for extrusion-based 3D printing of concrete is that the issue of cold joints should be handled with care. Their formation and consequence depend on many factors and can change substantially when a process is scaled up and the contour length increases substantially [30], although other parameters such as nozzle geometry, printing speed, layer height and material properties can play a role and be engineered to mitigate this. Variations of drying conditions during production will increase the variability of the interface quality. Results presented in this study concern samples that had 7 days of wet curing, which most probably attenuates the extent of cold joint formation with respect to what could be expected in production. Finally, the question of the structure (or rather layer) orientation with respect to chloride ingress will also play an important role, as well of course as the exposure conditions.
